# Drug-induced retinal vein occlusion: a disproportionality analysis from the FDA adverse event reporting system (2004–2023)

**DOI:** 10.3389/fphar.2024.1480269

**Published:** 2024-12-13

**Authors:** Xiao-Dong Chen, Kun-Hong Xiao, Chao-Bing Zhou

**Affiliations:** ^1^ Department of Ophthalmology, Hui’an County Hospital, Quanzhou, Fujian, China; ^2^ Eye Institute of Xiamen University, School of Medicine, Xiamen University, Xiamen, Fujian, China; ^3^ Department of Ophthalmology and Optometry, Fujian Medical University, Fuzhou, China; ^4^ Department of Ophthalmology, The Second Affiliated Hospital of Fujian Medical University, Quanzhou, Fujian, China

**Keywords:** retinal vein occlusion, FDA adverse event reporting system, disproportionality analysis, pharmacovigilance, adverse events

## Abstract

**Introduction:**

Retinal vein occlusion (RVO) often causes irreversible visual impairment, making early prevention crucial. This study aims to identify associations between different medications and RVO and provide information for clinical practice.

**Method:**

This study included reports of RVO from the FDA Adverse Event Reporting System (FAERS) database from the first quarter (Q1) of 2004 to the fourth quarter (Q4) of 2023. The reported drugs were analyzed for adverse drug reaction (ADR) signals using four disproportionality algorithms. Kaplan-Meier curves and median time to onset were used to evaluate the drugs.

**Results:**

From 2004 to 2023, the FAERS database recorded 6,151 reports associated with RVO. Disproportionality analyses identified 25 drugs significantly associated with RVO. Mirabegron showed the highest risk signal, followed by Raloxifene, Tadalafil, Fingolimod, and Bimatoprost. These high-risk drugs are distributed across different therapeutic areas, including urogenital system and sex hormones, ophthalmic drugs, nervous system drugs, musculoskeletal system drugs, anti-tumor and immune-modulating drugs, and anti-parasitic drugs. Specific drug targets such as adrenergic receptor agonists, hormone regulators, and PDE5 inhibitors were identified as high risk. Ophthalmic drugs exhibited the longest median time to adverse ocular reactions at 532.01 days, followed by anti-parasitic drugs, nervous system drugs, urogenital system and sex hormone drugs, anti-tumor and immune-modulating drugs, and musculoskeletal system drugs.

**Conclusion:**

This study provides an overview of drug-induced RVO, identifying potential culprit drugs and their distribution characteristics. These findings enhance understanding of medication safety and help optimize clinical practice.

## 1 Introduction

Retinal vein occlusion (RVO) is one of the most common vision-threatening eye diseases, second only to diabetic retinopathy (Khayat et al., 2018). The pathogenesis of RVO is complex, involving endothelial dysfunction, hemodynamic alterations, inflammatory responses, and genetic factors (Noma et al., 2020). RVO includes central retinal vein occlusion and branch retinal vein occlusion, both of which can lead to significant vision loss if not managed promptly (Kim et al., 2024). Early identification and prevention of risk factors are crucial to reduce the incidence of RVO and its resultant irreversible vision loss (Schmidt-Erfurth et al., 2019).

In recent years, with the widespread use of medications and the continuous introduction of new drugs, drug-induced adverse reactions have gradually garnered attention (Angamo et al., 2016). Although some case reports and small-scale studies have suggested that certain drugs may increase the risk of RVO (Lee et al., 2021; Etminan et al., 2022; Chen et al., 2023), there is a lack of large-scale systematic studies to assess the relationship between different drugs and RVO. This knowledge gap not only hinders clinicians’ decision-making when prescribing medications but also limits comprehensive drug safety evaluations. The Food and Drug Administration (FDA) Adverse Event Reporting System (FAERS) is a critical tool for drug safety monitoring, collecting reports of drug-related adverse events from around the world. By analyzing data from the FAERS database, potential associations between drugs and adverse events can be identified, providing valuable insights for clinical drug safety. Previous researchers have utilized this database to study drug-induced tooth discoloration (Wang et al., 2023), drug-induced allergic reactions (Yu et al., 2021), and drug induced acute pancreatitis (Li et al., 2024), offering references for clinical decision-making.

This study aims to utilize data from the FAERS database from 2004 to 2023 to evaluate the risk of drug-induced RVO through disproportionality analysis. We seek to investigate the association strength between different drugs and RVO and to conduct a more in-depth assessment of the induction time by these drugs. The goal is to provide clinicians with more comprehensive safety information when prescribing medications and to formulate more precise drug usage guidelines, ultimately reducing the risk.

## 2 Methods

### 2.1 Data source and study population

This study utilized data from the FAERS database, covering reports submitted from Q1 2004 through Q4 2023. FAERS includes mandatory reports from pharmaceutical companies and voluntary submissions from healthcare professionals (physicians and pharmacists), patients, and consumers. The dataset comprises demographic details, drug information, adverse event descriptions, therapy initiation and discontinuation dates, and treatment indications, all coded according to the Medical Dictionary for Regulatory Activities (MedDRA). The breadth of FAERS data provides a robust basis for drug safety signal detection (Li et al., 2024; Sakaeda et al., 2013). To ensure data reliability, we focused on reports submitted by healthcare professionals flagged as “Primary Suspect.”

### 2.2 Data selection and processing

RVO cases were identified using the MedDRA Preferred Term code 10038907. An initial dataset of 6,151 reports from healthcare professionals, patients, and consumers was reviewed. To enhance data accuracy and eliminate duplicates, we followed FDA guidelines, sorting entries by PRIMARYID, CASEID, and FDA_DT, retaining the most recent report with the highest PRIMARYID for entries with identical CASEID and FDA_DT values (Yin et al., 2022; Zhao and Tao, 2024). This process yielded 2,304 unique cases. Further analysis focused exclusively on reports from healthcare professionals and was restricted to drugs linked to at least three reported RVO cases. Additionally, we assigned Anatomical Therapeutic Chemical (ATC) codes to each drug to group and analyzed all RVO cases related to each active substance.

### 2.3 Signal detection methods and statistical analysis

To assess drug associations with RVO, we employed four established signal detection algorithms: Reporting Odds Ratio (ROR), Proportional Reporting Ratio (PRR), Bayesian Confidence Propagation Neural Network (BCPNN), and Multi-Item Gamma Poisson Shrinker (MGPS). The criteria for signal identification were: (1) ROR: a ≥ 3 and 95% CI lower > 1; (2) PRR: a ≥ 3 and 95% CI lower > 1; (3) BCPNN: IC_025_ > 0; and (4) MGPS: EBGM_05_ > 2 and a > 0 (Tables 1, 2). Meeting the thresholds across all four methods indicates a potential drug-event relationship (Wu et al., 2024a; Wu et al., 2024b; Wu et al., 2024c). Each method has specific strengths: ROR corrects for underreporting biases, while PRR provides enhanced specificity by comparing the reporting rate of a drug-event combination to others. BCPNN, based on Bayesian principles, integrates data from diverse sources and supports cross-validation, enhancing signal robustness. MGPS excels at identifying rare signals and managing sparse data (Jiang et al., 2024). By combining these methods, we maximized detection coverage and validated findings from multiple perspectives, strengthening the robustness of drug-RVO association assessments. All drug names were standardized using generic and brand names from the DrugBank database (Wishart et al., 2018). Statistical analyses were conducted using R (version 4.2.2), SPSS (version 26.0), and GraphPad Prism (version 10.1.2), with statistical significance set at *p* < 0.05. Further analyses examined dosage patterns and time to onset for drugs with positive signals.

**TABLE 1:** Four-grid table of disproportionality analysis method.

Item	Target adverse events	All other adverse events	Total
Target drugs	a	b	a+b
All other drugs	c	d	c + d
Total	a+c	b + d	a+b + c + d

Notes: A contingency table for the calculation formula of the proportion imbalance analysis.

**TABLE 2:** Principle of dis-proportionality measure and standard of signal detection.

Methods	Calculation formula	Inclusion standard of positive signal
ROR	$\text{ROR}=\frac{\left(a/c\right)}{\left(b/d\right)}$	a≥3 and 95%CI > 1
	$\text{SE}\left(\ln\text{ROR}\right)=\sqrt{\left(\frac{1}{a}+\frac{1}{b}+\frac{1}{c}+\frac{1}{d}\right)}$	
	$95\%\text{CI}=e^{\ln\left(\text{ROR}\right)\pm 1.96\sqrt{\frac{1}{a}+\frac{1}{b}+\frac{1}{c}+\frac{1}{d}}}$	
PRR	$\text{PRR}=\frac{a/\left(a+b\right)}{c/\left(c+d\right)}$	a≥3 and 95%CI > 1
	$\text{SE}\left(\ln\text{PRR}\right)=\sqrt{\left(\frac{1}{a}-\frac{1}{a+b}+\frac{1}{c}-\frac{1}{c+d}\right)}$	
	$95\%\text{CI}=e^{\ln\left(\text{PRR}\right)\pm 1.96\sqrt{\left(\frac{1}{a}-\frac{1}{a+b}+\frac{1}{c}-\frac{1}{c+d}\right)}}$	
BCPNN	$\text{IC}=\log_{2}\frac{a\left(a+b+c+d\right)}{\left(a+b\right)\left(a+c\right)}$	1) No Signal (−): IC_025_ ≤ 0 2) Low Signal (+):0<IC_025_ ≤ 1.5 3) Medium Signal (++):1.5<IC_025_ ≤ 3 4) High Signal (+++): IC_025_ > 3
	$E\left(\text{IC}\right)=\log_{2}\frac{\left(a+\gamma 11\right)\left(a+b+c+d+\alpha \right)\left(a+b+c+d+\beta \right)}{\left(a+b+c+d+\gamma \right)\left(a+b+\alpha 1\right)\left(a+c+\beta 1\right)}$	
	$\left.V\left(\text{IC}\right)=\frac{1}{{\left(\ln2\right)}^{2}}\{\left[\frac{\left(a+b+c+d\right)-a+\gamma -\gamma 11}{\left(a+\gamma 11\right)\left(1+a+b+c+d+\gamma \right)}\right]+\left[\frac{\left(a+b+c+d\right)-\left(a+b\right)+a-\alpha 1}{\left(a+b+\alpha 1\right)\left(1+a+b+c+d+\alpha \right)}\right]+\left[\frac{\left(a+b+c+d\right)-\left(a+c\right)+\beta -\beta 1}{\left(a+c+\beta 1\right)\left(1+a+b+c+d+\beta \right)}\right]\right\}$	
	$\gamma =\gamma 11\frac{\left(a+b+c+d+\alpha \right)\left(a+b+c+d+\beta \right)}{\left(a+b+\alpha 1\right)\left(a+c+\beta 1\right)}$	
	$\text{IC}-2\text{SD}=E\left(\text{IC}\right)-2\sqrt{V\left(\text{IC}\right)}$	
	Where α1 = β1 = 1; α = β = 2; γ11=1	
MGPS	$\text{EBGM}=\frac{a\left(a+b+c+d\right)}{\left(a+c\right)\left(a+b\right)}$	EBGM_05_ > 2 and a>0
	$\text{EBGM}05=e^{\ln\left(\text{EBGM}\right)-\sqrt[{2}]{1.64\left(\frac{1}{a}+\frac{1}{b}+\frac{1}{c}+\frac{1}{d}\right)}}$	

Abbreviation: ROR, reporting odds ratio; PRR, proportional reported ratio; BCPNN, bayesian confidence propagation neural network; MGPS, multi-item gamma poisson shrinker; CI, confidence interval; IC, information component.

### 2.4 Search strategy and terminology classification

RVO classification followed MedDRA standards, with the hierarchy structured as follows: System Organ Class (SOC): Eye Disorders; High-Level Group Term (HLGT): Retinal, Choroidal, and Vitreous Hemorrhages and Vascular Disorders; High-Level Term (HLT): Retinal Bleeding and Vascular Disorders (excluding Retinopathy); Preferred Term (PT): Retinal Vein Occlusion (Mozzicato, 2007; Brown et al., 1999). We used a narrow-scope standardized MedDRA query (SMQ) to ensure specificity, focusing on reports marked as “Primary Suspect” and submitted by healthcare professionals, minimizing irrelevant entries and enhancing data accuracy (Wu et al., 2024a; Li et al., 2023).

## 3 Results

### 3.1 Basic information on adverse events of retinal vein occlusion induced by drugs

**FIGURE 1:**
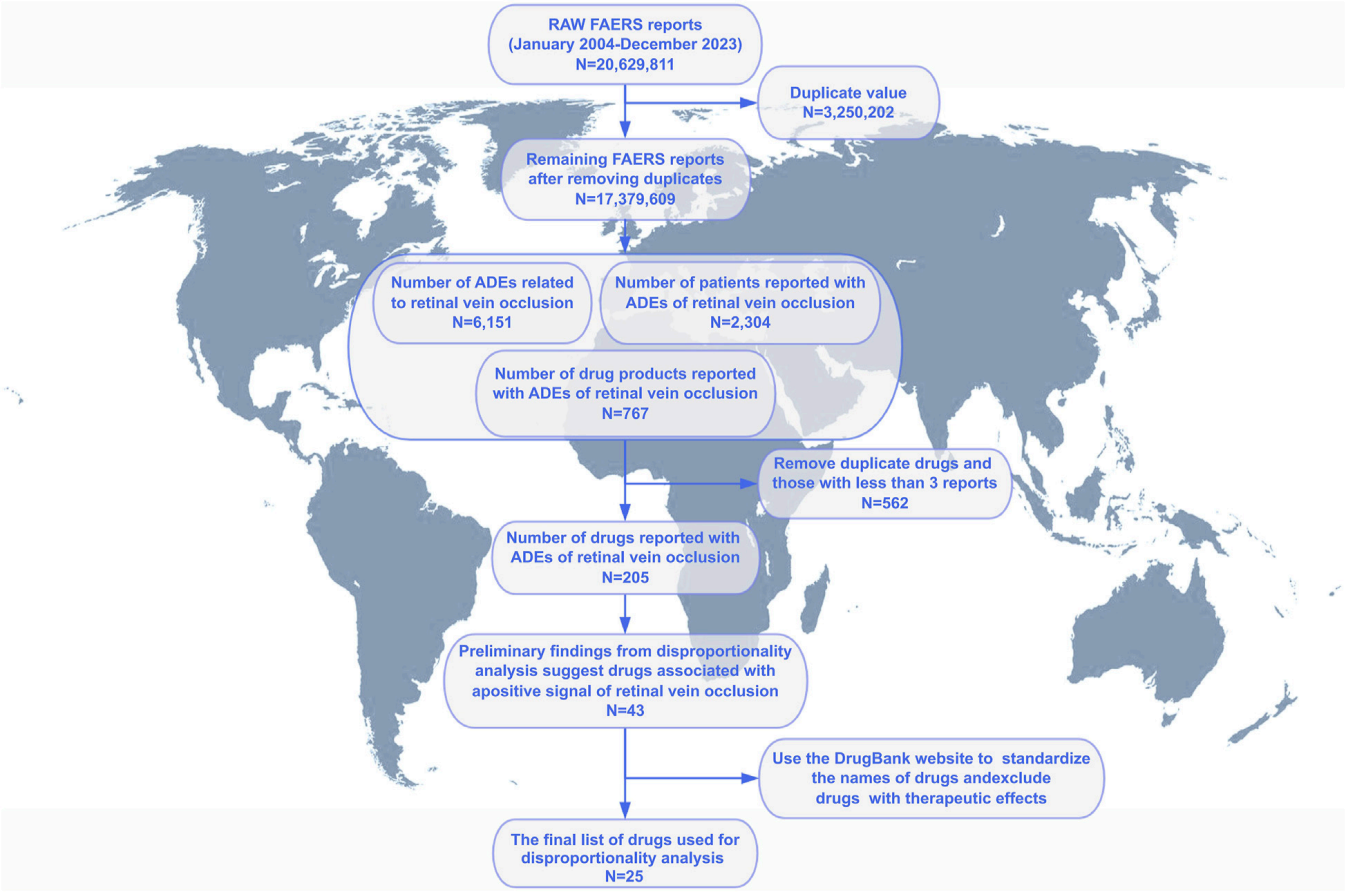
Flowchart of patient selection and data cleaning for drug-induced retinal vein occlusion in the FAERS database. Notes: Process of screening to obtain information on patients with retinal vein occlusion and their medication use from the FAERS database.

**FIGURE 2:**
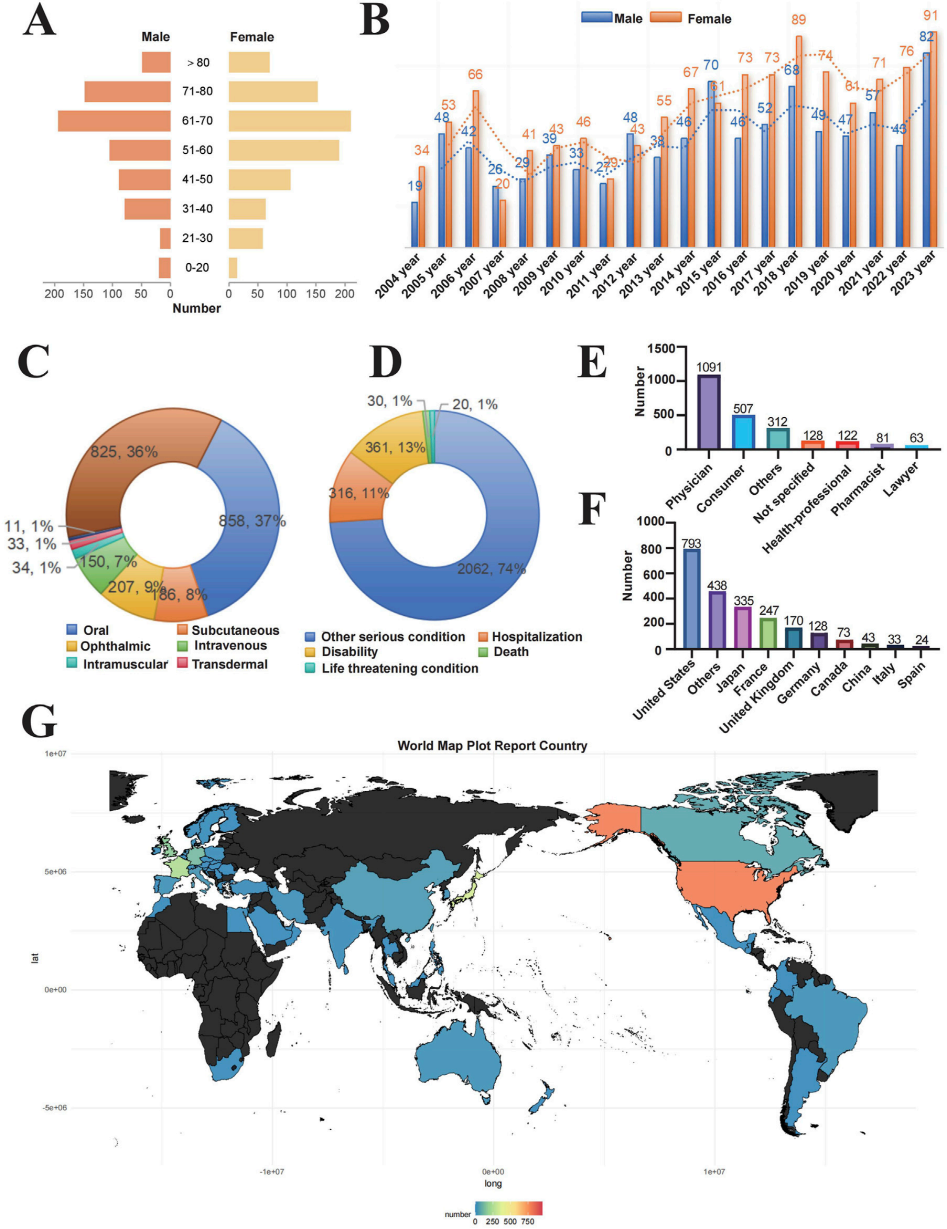
Distribution of baseline data for patients reporting adverse events of retinal vein occlusion in the FAERS database. Notes: Baseline characteristics of 2,304 patients with drug-induced RVO **(A)** Age and gender distribution of patients **(B)** Temporal trend of reported cases **(C)** Routes of administration **(D)** Patient outcomes **(E)** Occupational distribution of reporters **(F)** Number of reports by country **(G)** Geographic distribution of reports.

From January 2004 to December 2023, we analyzed a total of 20, 629, 811 adverse event reports from the FAERS database. Among these, 6,151 reports were related to RVO, encompassing 2,304 unique patients and 767 drug products. After deduplication and excluding drugs with fewer than three reports, 205 drugs were retained for analysis. Initial disproportionality analyses identified positive signals for 43 drugs. Following the standardization of drug names using the DrugBank database and excluding drugs with known therapeutic effects, a final list of 25 drugs was selected for further analysis (Figure 1). Patient demographics provided important context: the mean age was 58.8 ± 16.9 years, and the mean weight was 70.5 ± 21.2 kg. The most common route of administration was oral (37%), followed by subcutaneous (36%) and ophthalmic (9%) routes. Most adverse events were classified as “other serious conditions” (74%), with disability and hospitalization accounting for 13% and 11%, respectively. Death and life-threatening events each comprised 1%. In terms of report sources, the majority were submitted by physicians (47%), followed by consumers (22%). Geographically, the highest number of reports originated from the United States (793), followed by Japan (438), Germany (335), France (247), and the United Kingdom (170). Over the study period, the annual number of RVO-related ADE reports showed a fluctuating upward trend, with a comparable distribution of reports between male and female patients each year (Figure 2 and Table 3).

**TABLE 3:** Baseline data of retinal vein occlusion patients reported in the FAERS database.

Variables	Data presentation	Value
Age (year)	Mean ± SD	58.8 ± 16.9
	Median (Q1, Q3)	61 (49,71)
Weight (kg)	Mean ± SD	70.5 ± 21.2
	Median (Q1, Q3)	69 (56,82)
Gender		
Female	n (%)	1,166 (50.6%)
Male	n (%)	909 (39.5%)
Unknown	n (%)	229 (9.9%)
Outcome		
Other Serious (Important Medical Event)	n (%)	2062 (73.9%)
Disability	n (%)	361 (12.9%)
Hospitalization- Initial or Prolonged	n (%)	316 (11.3%)
Death	n (%)	30 (1.1%)
Life threatening condition	n (%)	20 (0.7%)
Country		
United States	n (%)	793 (34.4%)
Others	n (%)	458 (19.9%)
Japan	n (%)	335 (14.5%)
France	n (%)	247 (10.7%)
United Kingdom	n (%)	170 (7.4%)
Germany	n (%)	128 (5.6%)
Canada	n (%)	73 (3.2%)
China	n (%)	43 (1.9%)
Italy	n (%)	33 (1.4%)
Spain	n (%)	24 (1.0%)

Notes: Continuous numerical variables are expressed as mean ± standard deviation, and categorical variables are presented as n (%). Abbreviation: Mean ± SD, Mean ± Standard Deviation; Q1, Q3, First Quartile (25th Percentile), Third Quartile (75th Percentile); n (%), Number (Percentage).

### 3.2 Disproportionality analysis for identified positive risk signals

**FIGURE 3:**
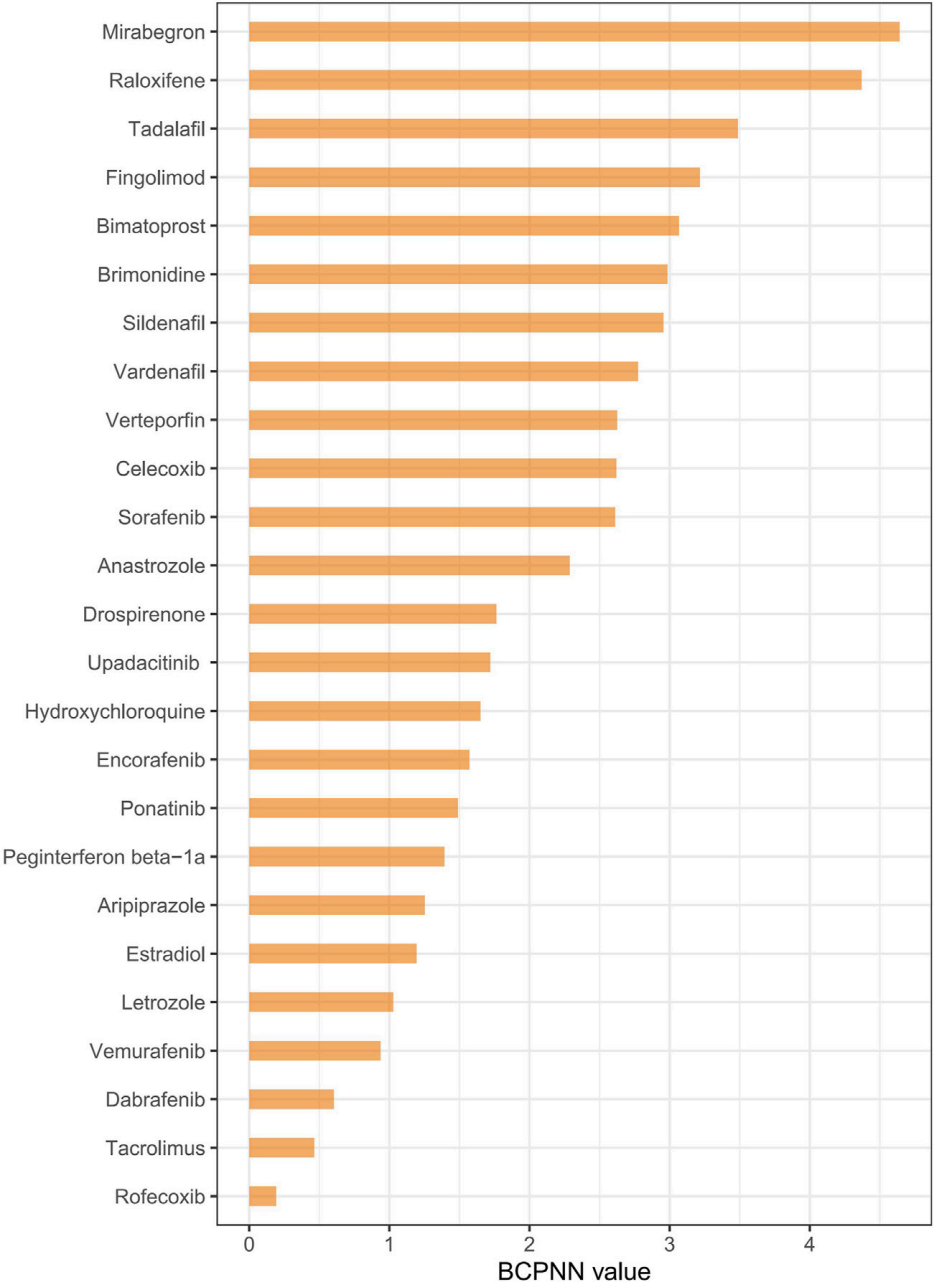
Ranking drug risk based on the BCPNN algorithm. Notes: Ranking drugs based on their risk of causing retinal vein occlusion using the BCPNN algorithm.

Disproportionality analysis, conducted using four algorithms (Table 4), identified 25 drugs with positive risk signals for RVO. The most frequently reported drugs, presented by their generic names, included sildenafil (n = 47) and rofecoxib (n = 47), followed by raloxifene (n = 26), tadalafil (n = 14), drospirenone (n = 11), and vemurafenib (n = 10). To further evaluate drug-associated risks, the BCPNN algorithm was applied, categorizing risk levels based on predefined thresholds: a BCPNN value between 0 and 1.5 indicates a low risk of drug-related adverse events, values between 1.5 and three denote a moderate risk, and values above three signify a high risk (Zhao et al., 2023). Using this approach, the drugs with the highest BCPNN values were mirabegron (4.64), raloxifene (4.37), tadalafil (3.488), fingolimod (3.215), and bimatoprost (3.065), indicating a strong association with an increased risk of RVO. These results suggest the need for closer monitoring and further assessment of these high-risk drugs in clinical settings (Figure 3 and Supplementary Table S1).

**TABLE 4:** Statistical values and distribution of drug-induced retinal vein occlusion.

Drug	Number	Classification	ROR (95%Cl)	PRR (X^2^)	MGPS (95% CI lower)	BCPNN (95% CI lower)	*p*-Value
Mirabegron	3	Urogenital System and Sex Hormone Drugs	80.255 (25.750–250.135)	79.730 (232.663)	79.533 (30.722)	6.313 (4.640)	<0.001
Raloxifene	26	Urogenital System and Sex Hormone Drugs	67.511 (45.723–99.681)	67.147 (1,657.496)	65.708 (47.426)	6.038 (4.370)	<0.001
Tadalafil	14	Urogenital System and Sex Hormone Drugs	36.190 (21.350–61.343)	36.085 (472.024)	35.674 (22.940)	5.157 (3.488)	<0.001
Sildenafil	47	Urogenital System and Sex Hormone Drugs	25.685 (19.181–34.394)	25.634 (1,068.974)	24.665 (19.319)	4.624 (2.956)	<0.001
Vardenafil	3	Urogenital System and Sex Hormone Drugs	21.859 (7.033–67.944)	21.822 (59.458)	21.769 (8.428)	4.444 (2.775)	<0.001
Drospirenone	11	Urogenital System and Sex Hormone Drugs	10.883 (6.009–19.711)	10.874 (97.726)	10.873 (6.560)	3.431 (1.763)	<0.001
Estradiol	6	Urogenital System and Sex Hormone Drugs	7.302 (3.273–16.291)	7.298 (32.451)	7.267 (3.713)	2.861 (1.193)	<0.001
Bimatoprost	4	Sensory Organ Drugs	26.757 (10.015–71.487)	26.700 (98.626)	26.614 (11.695)	4.734 (3.065)	<0.001
Brimonidine	3	Sensory Organ Drugs	25.307 (8.140–78.671)	25.256 (69.716)	25.195 (9.753)	4.655 (2.985)	<0.001
Verteporfin	4	Sensory Organ Drugs	19.716 (7.382–52.662)	19.686 (70.715)	19.623 (8.625)	4.294 (2.626)	<0.001
Aripiprazole	5	Nervous System Drugs	7.608 (3.160–18.317)	7.604 (28.558)	7.576 (3.632)	2.921 (1.253)	<0.001
Celecoxib	4	Musculoskeletal System Drugs	19.620 (7.346–52.404)	19.590 (70.333)	19.528 (8.583)	4.287 (2.618)	<0.001
Rofecoxib	47	Musculoskeletal System Drugs	3.734 (2.789–4.999)	3.733 (90.354)	3.626 (2.840)	1.858 (0.190)	<0.001
Fingolimod	6	Antitumor and Immunomodulating Drugs	29.746 (13.324–66.411)	29.675 (165.433)	29.531 (15.081)	4.884 (3.215)	<0.001
Sorafenib	4	Antitumor and Immunomodulating Drugs	19.485 (7.295–52.044)	19.455 (69.798)	19.394 (8.524)	4.278 (2.609)	<0.001
Anastrozole	7	Antitumor and Immunomodulating Drugs	15.592 (7.413–32.793)	15.573 (94.908)	15.487 (8.314)	3.953 (2.285)	<0.001
Upadacitinib	8	Antitumor and Immunomodulating Drugs	10.534 (5.254–21.120)	10.526 (68.513)	10.462 (5.846)	3.387 (1.719)	<0.001
Encorafenib	3	Antitumor and Immunomodulating Drugs	9.469 (3.048–29.416)	9.463 (22.650)	9.441 (3.657)	3.239 (1.570)	<0.001
Ponatinib	7	Antitumor and Immunomodulating Drugs	8.959 (4.260–18.838)	8.953 (49.166)	8.906 (4.782)	3.155 (1.487)	<0.001
Peginterferon beta-1a	3	Antitumor and Immunomodulating Drugs	8.365 (2.693–25.985)	8.360 (19.393)	8.342 (3.231)	3.060 (1.392)	<0.001
Letrozole	4	Antitumor and Immunomodulating Drugs	6.498 (2.434–17.347)	6.495 (18.536)	6.477 (2.848)	2.695 (1.027)	<0.001
Vemurafenib	10	Antitumor and Immunomodulating Drugs	6.128 (3.288–11.421)	6.125 (42.532)	6.083 (3.613)	2.605 (0.937)	<0.001
Dabrafenib	4	Antitumor and Immunomodulating Drugs	4.838 (1.812–12.914)	4.836 (12.133)	4.824 (2.121)	2.270 (0.602)	<0.005
Tacrolimus	7	Antitumor and Immunomodulating Drugs	4.401 (2.093–9.254)	4.400 (18.286)	4.380 (2.352)	2.131 (0.463)	<0.001
Hydroxychloroquine	3	Antiparasitic Drugs	10.005 (3.221–31.080)	9.997 (24.233)	9.975 (3.864)	3.318 (1.650)	<0.001

Note: The *p*-value represents the statistical test value from the chi-square test in the PRR, algorithm.

Abbreviation: ROR, reporting odds ratio; PRR, proportional reported ratio; BCPNN, bayesian confidence propagation neural network; MGPS, multi-item gamma poisson shrinker; CI, confidence interval.

### 3.3 Distribution of RVO risk drugs and time to onset of ocular adverse reactions

**FIGURE 4:**
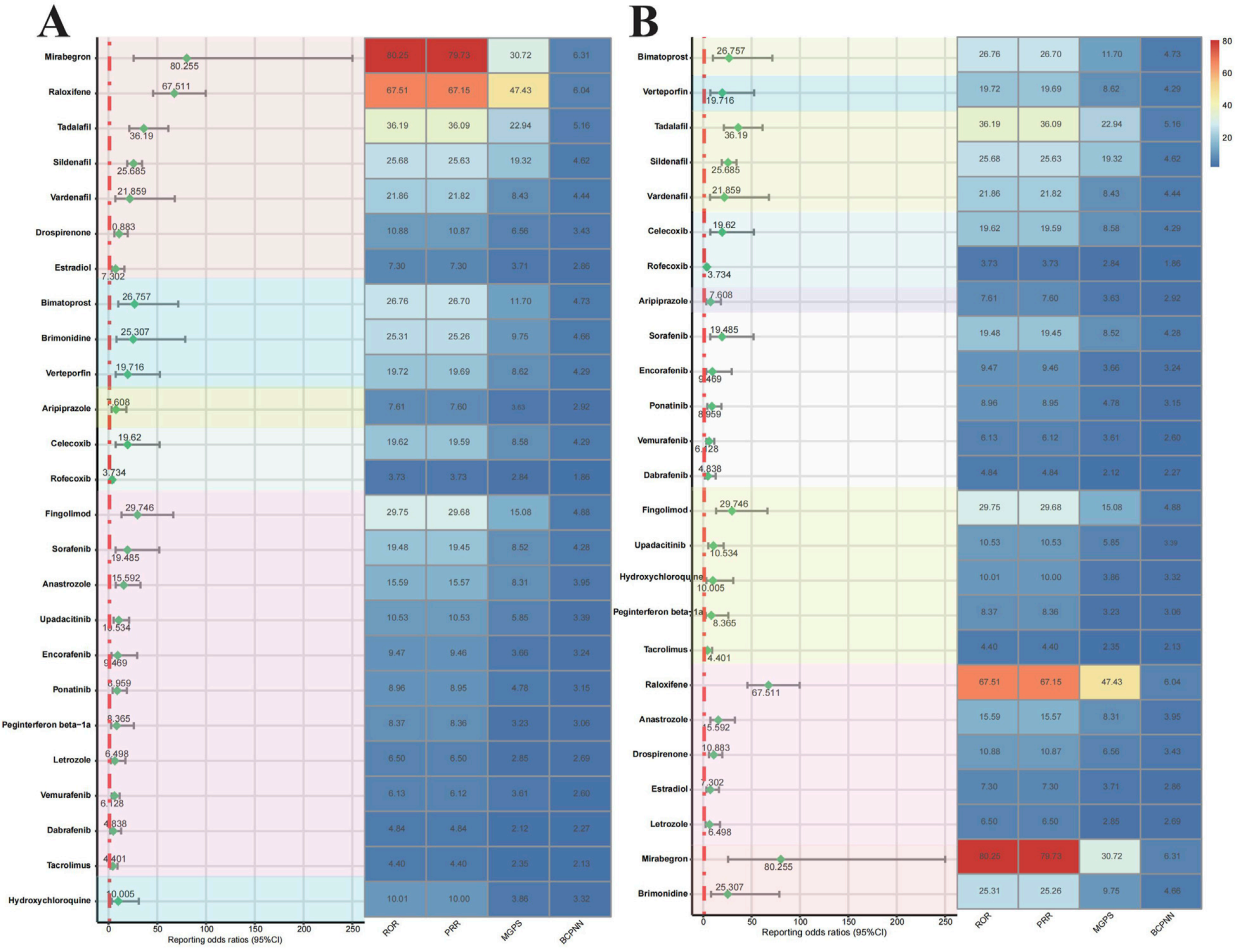
Forest plots and heat maps of drugs with positive signals for drug-induced retinal vein occlusion based on disproportionality analysis methods from the FAERS database. Notes: Disproportionality analysis of RVO risk drugs based on ATC classification **(A)** and target classification **(B)**. Abbreviation: ROR, reporting odds ratio; PRR, proportional reported ratio; BCPNN, Bayesian confidence propagation neural network; MGPS, multi-item gamma poisson shrinker. Abbreviation: BCPNN, Bayesian confidence propagation neural network.

**FIGURE 5:**
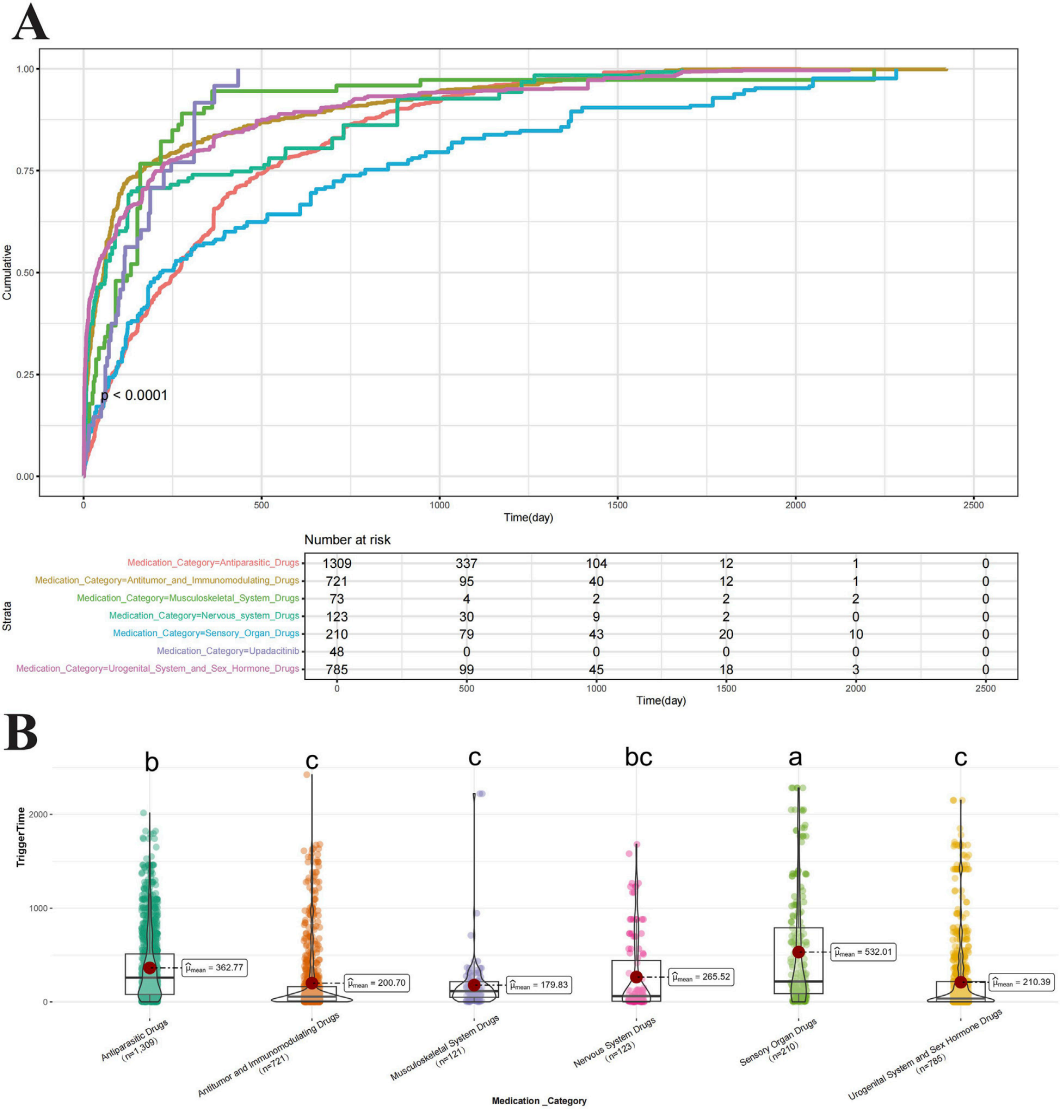
Cumulative risk curves for ocular adverse reactions by drug classification. Notes: The timeline of ocular adverse reactions induced by related drugs: **(A)** Kaplan-Meier curve **(B)** Median induction time. Statistical differences are labeled with letters; groups with the same letter indicate no significant difference.

We ranked the risk of drugs associated with RVO based on the ATC classification (Figure 4A). The risk of RVO induced by Urogenital System and Sex Hormone Drugs was higher than that of other drug categories. This category included Mirabegron (ROR = 80.255), Raloxifene (ROR = 67.511), Tadalafil (ROR = 36.19), Sildenafil (ROR = 25.685), Vardenafil (ROR = 21.859), Drospirenone (ROR = 10.883), and Estradiol (ROR = 7.302), with Mirabegron and Raloxifene being the most prominent. Ophthalmic drugs include Bimatoprost (ROR = 26.757), Brimonidine (ROR = 25.307), and Verteporfin (ROR = 19.716). Nervous System Drugs included Aripiprazole (ROR = 7.608). Musculoskeletal System Drugs included Celecoxib (ROR = 19.62) and Rofecoxib (ROR = 3.734). Antitumor and Immunomodulating Drugs include Fingolimod (ROR = 29.746), Sorafenib (ROR = 19.485), Anastrozole (ROR = 15.592), Upadacitinib (ROR = 10.534), Encorafenib (ROR = 9.469), Ponatinib (ROR = 8.959), Peginterferon beta-1a (ROR = 8.365), Letrozole (ROR = 6.498), Vemurafenib (ROR = 6.128), Dabrafenib (ROR = 4.838), and Tacrolimus (ROR = 4.401). Antiparasitic Drugs include Hydroxychloroquine (ROR = 10.005). Based on the identification of drugs by their target sites (Figure 4B), we found that Adrenergic Receptor Agonists (Mirabegron, Brimonidine), Hormone Modulators (Raloxifene, Anastrozole, Drospirenone, Estradiol, Letrozole), and PDE5 Inhibitors (Tadalafil, Sildenafil, Vardenafil) had a higher risk of inducing RVO. The Kaplan-Meier curves (Figure 5A) showed significant differences in adverse ocular reactions induction time across different drug categories (*p* < 0.0001). Specifically, the survival curves for Urogenital System and Sex Hormone Drugs, Nervous System Drugs, and Antitumor and Immunomodulating Drugs were steeper in the early stages, indicating faster induction of RVO. In contrast, the curve for Ophthalmic Drugs was more gradual, indicating a longer induction time. Moreover, the median adverse ocular reactions induction time (Figure 5B) analysis revealed that Ophthalmic Drugs have the longest induction time at 532.01 days, followed by Antiparasitic Drugs (362.77 days), Nervous System Drugs (265.52 days), Urogenital System and Sex Hormone Drugs (210.39 days), Antitumor and Immunomodulating Drugs (200.7 days), and Musculoskeletal System Drugs (179.83 days). For more details on the induction times for specific drugs, see Table 5.

**TABLE 5:** Trigger times for drug-induced ocular adverse reactions.

Drug	Median	Q1	Q3
Verteporfin	396	114.5	1,034
Tacrolimus	334	107	966
Hydroxychloroquine	259	80	512
Sildenafil	164	14	746
Mirabegron	163	51	900
Tadalafil	153	8	424
Brimonidine	152	63	511
Sorafenib	150	14	651
Celecoxib	142	35	159
Bimatoprost	129	67.5	190.5
Upadacitinib	113	61	230.25
Anastrozole	100	14	695
Vemurafenib	97.5	52	166.25
Ponatinib	96	72	1,021
Rofecoxib	90	29.5	204
Drospirenone	86	14	200
Aripiprazole	74	11.5	566.5
Vardenafil	71	10.5	165
Dabrafenib	51	13	62
Letrozole	37	13	103
Estradiol	32	7	154
Encorafenib	28	7	64
Peginterferon beta-1a	17	7	151
Fingolimod	4	3.75	120.5
Raloxifene	1	1	8

Notes: Time of onset of drug-induced ocular adverse reactions caused by the drug.

Abbreviations: Q1, First Quartile (25th Percentile); Q3, Third Quartile (75th Percentile).

## 4 Discussion

Vision loss caused by RVO is often irreversible, making early prevention crucial. This study systematically examined the relationship between various drugs and RVO using FAERS database. 25 drugs, including 6 ATC categories and 9 different drug targets were found statistically associated with the risk of RVO. Based on these findings, clinicians should regularly assess the medications prescribed to patients, especially those associated with an increased risk of RVO, and adjust treatment plans when necessary. Enhanced retinal health monitoring is recommended for patients on high-risk medications, especially those with vascular risk factors such as hypertension, diabetes, or cardiovascular conditions. When possible, clinicians should consider prescribing drugs with a lower risk of RVO and inform patients about the potential retinal risks associated with their medications, encouraging them to report any vision changes for early intervention. Through these, clinicians can effectively reduce the risk of vision loss related to RVO and improve patient outcomes.

Among these, mirabegron showed the highest risk of RVO. Mirabegron, a β3-adrenergic receptor agonist used to treat overactive bladder, has been noted for its efficacy in reducing incontinence and urinary frequency (Herschorn et al., 2020). Animal researches suggested mirabegron affects choroidal thickness and vascular responses to some extent (Topcuoglu and Aslan, 2021). Sui et al. (2019) found that it might induce cardiovascular diseases in atherosclerosis patients by activating brown adipose tissue-mediated lipolysis. Hypertension and atherosclerosis can directly damage the vascular endothelium, reducing the ability of endothelial cells to prevent thrombosis, and thus making RVO more likely to occur (Scott et al., 2020). Despite its significant efficacy and good tolerance in treating overactive bladder, the potential RVO risk of mirabegron warrants sufficient attention in clinical practice, especially for patients with pre-existing cardiovascular diseases or other vascular risk factors. Raloxifene, used to reduce the risk of invasive breast cancer in postmenopausal high-risk women, also increases the risk of venous thromboembolism in postmenopausal women (Mosca et al., 2009), which explains its role in RVO. Tadalafil, sildenafil, and vardenafil are selective and reversible PDE5 inhibitors used to treat erectile dysfunction and pulmonary arterial hypertension. Frequent use of PDE5Is has been shown to potentially increase the risk of serous retinal detachment, retinal vascular occlusion, and ischemic optic neuropathy (Etminan et al., 2022), which aligns with our study findings. High levels of estradiol may increase blood coagulability, leading to a higher risk of thrombosis (Coleman et al., 2023). This prothrombotic tendency could trigger occlusion in the retinal veins. Drospirenone, commonly used in oral contraceptives, has not yet been reported to be associated with RVO, but oral contraceptives containing drospirenone have a higher risk of venous thromboembolism (Larivée et al., 2017), which could be closely related to RVO.

In ophthalmic medications, bimatoprost, brimonidine, and verteporfin all showed relatively high ROR for RVO. However, prior studies have not reported a clear association between these drugs and RVO. Long-term use of these medications in individuals with conditions like glaucoma or AMD means that these patients are inherently at a higher risk of RVO due to their underlying ocular and systemic vascular risk factors (Weinstein et al., 2023). The longer induction times observed in our study further support this interpretation. Therefore, while it is essential for clinicians to monitor for potential adverse effects, the elevated RVO risk observed in our analysis should be interpreted within the context of these patients’ overall health profiles.

In our study, aripiprazole is the only neurological drug associated with an increased risk of RVO. Aripiprazole, an atypical antipsychotic that partially agonizes dopamine D2 receptors and serotonin 5-HT1A receptors while antagonizing 5-HT2A receptors, is used for schizophrenia and bipolar disorder (Findling et al., 2008). Faure et al. (2015) reported aripiprazole use might lead to choroidal retinopathy, impacting retinal pigment epithelium, consistent with our findings.

Antineoplastic and immunomodulatory agents also demonstrated a higher ROR for RVO. Fingolimod is an immunomodulator primarily used to treat relapsing forms of multiple sclerosis (MS) by inhibiting lymphocyte migration and reducing central nervous system inflammation (Cohen and Chun, 2011). Prior case reports have linked fingolimod treatment to sudden vision loss due to temporal superior branch RVO in MS patients, which aligns with our findings (Gallego-Pinazo et al., 2011). Sorafenib, a multi-target tyrosine kinase inhibitor, reduces tumor angiogenesis by inhibiting Raf kinase, VEGFR, and PDGFR, but its anti-angiogenic effects can lead to decreased retinal blood flow and vessel narrowing, increasing RVO risk (Liu et al., 2006). Previous studies have reported RVO associated with long-term sorafenib use, necessitating regular ophthalmic examinations (Szczepanik and Kęcik, 2012). Gaertner et al. (2014) noted sorafenib’s potential to cause retinal tear in some cancer patients. Anastrozole and letrozole, aromatase inhibitors, reduce estrogen synthesis, and low estrogen levels can lead to endothelial dysfunction, increasing thrombotic risk (Geisler et al., 2002; Somani et al., 2019). Almafreji et al. (2021) indicated anastrozole and letrozole might cause optic disc edema, macular edema, and uveitis, potentially leading to retinal hemorrhage or RVO. Encorafenib, vemurafenib, and dabrafenib, BRAF kinase inhibitors used for BRAF-mutant melanoma, are linked to uveitis and other retinal side effects (Grob et al., 2015; Liu et al., 2014). Similarly, peginterferon beta-1a, a long-acting interferon used for MS, modulates immune responses to reduce disease activity (Newsome et al., 2017). Interferons have been reported to cause endothelial damage and abnormal blood coagulation, thereby increasing the risk of venous occlusion (Jia et al., 2018). Ponatinib, a tyrosine kinase inhibitor for chronic myeloid leukemia (CML) and acute lymphoblastic leukemia (ALL), induces vascular toxicity, which can be permanent or transient, contributing to RVO (Herrmann, 2016). Upadacitinib, a selective JAK inhibitor used for rheumatoid arthritis and other autoimmune diseases, has limited studies on its retinal vascular effects. However, on the other hand, the specificity of JAK inhibitors may increase the risk of thromboembolic side effects, as blocking a single pathway can disrupt the balance between pro-thrombotic and anti-thrombotic activities (Kotyla et al., 2021). Tacrolimus, an immunosuppressant used to prevent organ transplant rejection, inhibits T-cell activation and immune response, but Jun et al. found it causes hypercoagulable states in ocular vessels, leading to central RVO and vision loss (Wu et al., 2018). Anti-inflammatory and analgesic medications such as rofecoxib and celecoxib were also found to be associated with RVO in our study. Rofecoxib and celecoxib, selective COX-2 inhibitors, reduce inflammation and pain by inhibiting cyclooxygenase-2, but they may induce prothrombotic effects under certain conditions (Mukherjee et al., 2001; Meyer et al., 2005). Patients susceptible to thrombosis might be at risk of ocular thrombotic events (Meyer et al., 2005). The antimalarial and autoimmune medication hydroxychloroquine was confirmed to have a significant association with RVO. Long-term hydroxychloroquine use is known to cause retinal toxicity, leading to paracentral scotomas and loss of photoreceptor inner and outer segments (Proano and Kimball, 2019). In the United Kingdom, long-term hydroxychloroquine and chloroquine users are advised to undergo retinal screening to detect potential ocular diseases (Yusuf et al., 2018). Our study further corroborates its role in RVO.

Simultaneously, we further evaluated the time course of ocular adverse reactions induced by different drug categories and found significant differences in trigger times among these categories. Specifically, Urogenital System and Sex Hormone Drugs and Nervous System Drugs tend to have shorter induction times for adverse reactions, suggesting that patients using these drugs may experience ocular issues at an earlier stage. Thus, more frequent and earlier ophthalmic follow-ups are warranted for these patients to promptly detect and manage potential adverse effects. In contrast, Sensory Organ Drugs exhibit longer induction times for adverse reactions, indicating that patients using these drugs may require less frequent monitoring of retinal vasculature, allowing for longer intervals between regular check-ups to ensure safety. This finding has important implications for developing clinical monitoring strategies. Personalized follow-up schedules based on the time characteristics of different drug categories could help optimize the use of medical resources and allow for timely intervention in high-risk early stages.

This study’s primary limitation lies in its inability to distinguish between ischemic and non-ischemic RVO subtypes, which are clinically distinct in severity and management (Hayreh, 2021). The FAERS database’s lack of granularity in this aspect constrains the precision of our findings. Moreover, the absence of visual acuity data hinders a comprehensive assessment of the extent and progression of vision loss associated with drug-induced RVO, limiting our understanding of its clinical implications. The voluntary nature of FAERS reporting also introduces potential biases, including underreporting or selective reporting of adverse events, which may impact the strength and clarity of observed associations. To enhance the reliability and depth of future analyses, integrating FAERS data with complementary sources, such as electronic health records (EHRs) and insurance claims, would provide a more robust and accurate dataset, mitigating these inherent biases (Montastruc et al., 2006).

Clinically, our findings emphasize that patients on medications with an elevated RVO risk—particularly older adults or those with existing vascular conditions—should be subject to regular vascular assessments and routine eye examinations to detect early signs of RVO and enable timely interventions. When feasible, switching to lower-risk alternative therapies should be considered. Educating patients to recognize early visual symptoms may also facilitate quicker diagnoses and treatment, potentially minimizing severe vision loss. Moving forward, future research should prioritize prospective cohort studies to validate these drug-RVO associations and elucidate underlying mechanisms. A targeted focus on high-risk populations will be critical for refining screening and management guidelines. By integrating diverse data sources, such as EHRs tied to clinical outcomes, we can achieve a more comprehensive understanding of drug-induced RVO, ultimately improving drug safety assessments and informing better clinical decisions.

## 5 Conclusion

This study provides a comprehensive analysis of drug-induced RVO, identifying potential causative drugs, examining their distribution patterns, and offering insights into drug targets. These findings not only enhance our understanding of medication safety but also provide crucial information for optimizing clinical practices. By highlighting high-risk drug categories and targets, this research underscores the importance of informed prescribing decisions and ongoing pharmacovigilance efforts in safeguarding patient ocular health.

## Data Availability

Publicly available datasets were analyzed in this study. These data can be found at: https://fis.fda.gov/extensions/FPD-QDE-FAERS/FPD-QDE-FAERS.html.

## References

[B1] Almafreji I. Smith C. Peck F. (2021). Review of the literature on ocular complications associated with aromatase inhibitor use. Cureus 13 (8), e17565. 10.7759/cureus.17565 34646621 PMC8482805

[B2] Angamo M. T. Chalmers L. Curtain C. M. Bereznicki L. R. E. (2016). Adverse-drug-reaction-related hospitalisations in developed and developing countries: a review of prevalence and contributing factors. Drug Saf. 39 (9), 847–857. 10.1007/s40264-016-0444-7 27449638

[B3] Brown E. G. Wood L. Wood S. (1999). The medical dictionary for regulatory activities (MedDRA). Drug Saf. 20 (2), 109–117. 10.2165/00002018-199920020-00002 10082069

[B4] Chen Y. Du Y. Qiu L. Zheng J. (2023). Central retinal vein occlusion with cerebral infarction secondary to anlotinib treatment: a case report and literature review. Front. Pharmacol. 14, 1188218. 10.3389/fphar.2023.1188218 37383723 PMC10293757

[B5] Cohen J. A. Chun J. (2011). Mechanisms of fingolimod’s efficacy and adverse effects in multiple sclerosis. Ann. Neurol. 69 (5), 759–777. 10.1002/ana.22426 21520239

[B6] Coleman J. R. Moore E. E. Schmitt L. Hansen K. Dow N. Freeman K. (2023). Estradiol provokes hypercoagulability and affects fibrin biology: a mechanistic exploration of sex dimorphisms in coagulation. J. Trauma Acute Care Surg. 94 (2), 179–186. 10.1097/TA.0000000000003822 36694329 PMC9881840

[B7] Etminan M. Sodhi M. Mikelberg F. S. Maberley D. (2022). Risk of ocular adverse events associated with use of phosphodiesterase 5 inhibitors in men in the US. JAMA Ophthalmol. 140 (5), 480–484. 10.1001/jamaophthalmol.2022.0663 35389459 PMC8990352

[B8] Faure C. Audo I. Zeitz C. Letessier J. B. Robert M. P. (2015). Aripiprazole-induced chorioretinopathy: multimodal imaging and electrophysiological features. Doc. Ophthalmol. 131 (1), 35–41. 10.1007/s10633-015-9494-x 25791769

[B9] Findling R. L. Robb A. Nyilas M. Forbes R. A. Jin N. Ivanova S. (2008). A multiple-center, randomized, double-blind, placebo-controlled study of oral aripiprazole for treatment of adolescents with schizophrenia. Am. J. Psychiatry 165 (11), 1432–1441. 10.1176/appi.ajp.2008.07061035 18765484

[B10] Gaertner K. M. Caldwell S. H. Rahma O. E. (2014). A case of retinal tear associated with use of sorafenib. Front. Oncol. 4, 196. 10.3389/fonc.2014.00196 25105094 PMC4109517

[B11] Gallego-Pinazo R. España-Gregori E. Casanova B. Pardo-López D. Díaz-Llopis M. (2011). Branch retinal vein occlusion during fingolimod treatment in a patient with multiple sclerosis. J. Neuroophthalmol. 31 (3), 292–293. 10.1097/WNO.0b013e31822bed20 21826025

[B12] Geisler J. Haynes B. Anker G. Dowsett M. Lønning P. E. (2002). Influence of letrozole and anastrozole on total body aromatization and plasma estrogen levels in postmenopausal breast cancer patients evaluated in a randomized, cross-over study. J. Clin. Oncol. 20 (3), 751–757. 10.1200/JCO.2002.20.3.751 11821457

[B13] Grob J. J. Amonkar M. M. Karaszewska B. Schachter J. Dummer R. Mackiewicz A. (2015). Comparison of dabrafenib and trametinib combination therapy with vemurafenib monotherapy on health-related quality of life in patients with unresectable or metastatic cutaneous BRAF Val600-mutation-positive melanoma (COMBI-v): results of a phase 3, open-label, randomised trial. Lancet Oncol. 16 (13), 1389–1398. 10.1016/S1470-2045(15)00087-X 26433819

[B14] Hayreh S. S. (2021). Photocoagulation for retinal vein occlusion. Prog. Retin Eye Res. 85, 100964. 10.1016/j.preteyeres.2021.100964 33713810

[B15] Herrmann J. (2016). Tyrosine kinase inhibitors and vascular toxicity: impetus for a classification system? Curr. Oncol. Rep. 18 (6), 33. 10.1007/s11912-016-0514-0 27099141

[B16] Herschorn S. Staskin D. Schermer C. R. Kristy R. M. Wagg A. (2020). Safety and tolerability results from the pillar study: a phase IV, double-blind, randomized, placebo-controlled study of mirabegron in patients ≥ 65 years with overactive bladder-wet. Drugs Aging 37 (9), 665–676. 10.1007/s40266-020-00783-w 32725584 PMC7473960

[B17] Jia H. Thelwell C. Dilger P. Bird C. Daniels S. Wadhwa M. (2018). Endothelial cell functions impaired by interferon *in vitro*: insights into the molecular mechanism of thrombotic microangiopathy associated with interferon therapy. Thromb. Res. 163, 105–116. 10.1016/j.thromres.2018.01.039 29407621

[B18] Jiang Y. Zhou L. Shen Y. Zhou Q. Ji Y. Zhu H. (2024). Safety assessment of Brexpiprazole: real-world adverse event analysis from the FAERS database. J. Affect Disord. 346, 223–229. 10.1016/j.jad.2023.11.025 37956832

[B19] Khayat M. Williams M. Lois N. (2018). Ischemic retinal vein occlusion: characterizing the more severe spectrum of retinal vein occlusion. Surv. Ophthalmol. 63 (6), 816–850. 10.1016/j.survophthal.2018.04.005 29705175

[B20] Kim M. S. Nam S. Lee S. U. Park S. J. Woo S. J. Lee J. (2024). Moyamoya disease increased the risk of retinal vascular occlusion: a nationwide cohort study in korea. Ophthalmol. Retina (24), S2468–S6530. Published online October 21. 10.1016/j.oret.2024.10.013 39442651

[B21] Kotyla P. J. Engelmann M. Giemza-Stokłosa J. Wnuk B. Islam M. A. (2021). Thromboembolic adverse drug reactions in janus kinase (JAK) inhibitors: does the inhibitor specificity play a role? Int. J. Mol. Sci. 22 (5), 2449. 10.3390/ijms22052449 33671049 PMC7957632

[B22] Larivée N. Suissa S. Coulombe J. Tagalakis V. Filion K. B. (2017). Drospirenone-containing oral contraceptive pills and the risk of venous thromboembolism: an assessment of risk in first-time users and restarters. Drug Saf. 40 (7), 583–596. 10.1007/s40264-017-0525-2 28382493

[B23] Lee M. K. Kim B. Han K. Lee J. H. Kim M. Kim M. K. (2021). Sodium-Glucose cotransporter 2 inhibitors and risk of retinal vein occlusion among patients with type 2 diabetes: a propensity score-matched cohort study. Diabetes Care. Epub of print. 10.2337/dc20-3133 34301735

[B24] Li D. Gou J. Zhu J. Zhang T. Liu F. Zhang D. (2023). Severe cutaneous adverse reactions to drugs: a real-world pharmacovigilance study using the FDA Adverse Event Reporting System database. Front. Pharmacol. 14, 1117391. 10.3389/fphar.2023.1117391 37081961 PMC10110972

[B25] Li D. Wang H. Qin C. Du D. Wang Y. Du Q. (2024). Drug-induced acute pancreatitis: a real-world pharmacovigilance study using the FDA adverse event reporting system database. Clin. Pharmacol. Ther. 115 (3), 535–544. 10.1002/cpt.3139 38069538

[B26] Liu C. Y. Francis J. H. Brodie S. E. Marr B. Pulido J. S. Marmor M. F. (2014). Retinal toxicities of cancer therapy drugs: biologics, small molecule inhibitors, and chemotherapies. Retina 34 (7), 1261–1280. 10.1097/IAE.0000000000000242 24949716

[B27] Liu L. Cao Y. Chen C. Zhang X. McNabola A. Wilkie D. (2006). Sorafenib blocks the RAF/MEK/ERK pathway, inhibits tumor angiogenesis, and induces tumor cell apoptosis in hepatocellular carcinoma model PLC/PRF/5. Cancer Res. 66 (24), 11851–11858. 10.1158/0008-5472.CAN-06-1377 17178882

[B28] Meyer C. H. Schmidt J. C. Rodrigues E. B. Mennel S. (2005). Risk of retinal vein occlusions in patients treated with rofecoxib (vioxx). Ophthalmologica 219 (4), 243–247. 10.1159/000085735 16088245

[B29] Montastruc J. L. Sommet A. Lacroix I. Olivier P. Durrieu G. Damase-Michel C. (2006). Pharmacovigilance for evaluating adverse drug reactions: value, organization, and methods. Jt. Bone Spine 73 (6), 629–632. 10.1016/j.jbspin.2006.09.002 17110152

[B30] Mosca L. Grady D. Barrett-Connor E. Collins P. Wenger N. Abramson B. L. (2009). Effect of raloxifene on stroke and venous thromboembolism according to subgroups in postmenopausal women at increased risk of coronary heart disease. Stroke 40 (1), 147–155. 10.1161/STROKEAHA.108.518621 18948611 PMC3559135

[B31] Mozzicato P. (2007). Standardised MedDRA queries: their role in signal detection. Drug Saf. 30 (7), 617–619. 10.2165/00002018-200730070-00009 17604415

[B32] Mukherjee D. Nissen S. E. Topol E. J. (2001). Risk of cardiovascular events associated with selective COX-2 inhibitors. JAMA 286 (8), 954–959. 10.1001/jama.286.8.954 11509060

[B33] Newsome S. D. Kieseier B. C. Liu S. You X. Kinter E. Hung S. (2017). Peginterferon beta-1a reduces disability worsening in relapsing-remitting multiple sclerosis: 2-year results from ADVANCE. Ther. Adv. Neurol. Disord. 10 (1), 41–50. 10.1177/1756285616676065 28450894 PMC5400156

[B34] Noma H. Yasuda K. Shimura M. (2020). Cytokines and pathogenesis of central retinal vein occlusion. J. Clin. Med. 9 (11), 3457. 10.3390/jcm9113457 33121094 PMC7692731

[B35] Proano C. Kimball G. P. (2019). Hydroxychloroquine retinal toxicity. N. Engl. J. Med. 380 (17), e27. 10.1056/NEJMicm1304542 31018072

[B36] Sakaeda T. Tamon A. Kadoyama K. Okuno Y. (2013). Data mining of the public version of the FDA adverse event reporting system. Int. J. Med. Sci. 10 (7), 796–803. 10.7150/ijms.6048 23794943 PMC3689877

[B37] Schmidt-Erfurth U. Garcia-Arumi J. Gerendas B. S. Midena E. Sivaprasad S. Tadayoni R. (2019). Guidelines for the management of retinal vein occlusion by the European society of Retina specialists (EURETINA). Ophthalmologica 242 (3), 123–162. 10.1159/000502041 31412332

[B38] Scott I. U. Campochiaro P. A. Newman N. J. Biousse V. (2020). Retinal vascular occlusions. Lancet 396 (10266), 1927–1940. 10.1016/S0140-6736(20)31559-2 33308475 PMC9546635

[B39] Somani Y. B. Pawelczyk J. A. De Souza M. J. Kris-Etherton P. M. Proctor D. N. (2019). Aging women and their endothelium: probing the relative role of estrogen on vasodilator function. Am. J. Physiol. Heart Circ. Physiol. 317 (2), H395–H404. 10.1152/ajpheart.00430.2018 31173499 PMC6732482

[B40] Sui W. Li H. Yang Y. Jing X. Xue F. Cheng J. (2019). Bladder drug mirabegron exacerbates atherosclerosis through activation of brown fat-mediated lipolysis. Proc. Natl. Acad. Sci. U. S. A. 116 (22), 10937–10942. 10.1073/pnas.1901655116 31085638 PMC6561204

[B41] Szczepanik S. Kęcik D. (2012). Bilateral central retinal vein occlusion in a patient with disseminated metastatic renal cell carcinoma treated with sorafenib. Retin Cases Brief. Rep. 6 (2), 148–150. 10.1097/ICB.0b013e3182160965 25390947

[B42] Topcuoglu M. Aslan F. (2021). Evaluation of the effect of a novel β3-adrenergic agonist on choroidal vascularity. Invest. Ophthalmol. Vis. Sci. 62 (9), 17. 10.1167/iovs.62.9.17 PMC8287045 34241623

[B43] Wang J. Zou D. Li Y. Liu P. Guo C. (2023). Drug-induced tooth discoloration: an analysis of the US food and drug administration adverse event reporting system. Front. Pharmacol. 14, 1161728. 10.3389/fphar.2023.1161728 37124229 PMC10133538

[B44] Weinstein O. Kridin M. Kridin K. Mann O. Cohen A. D. Zloto O. (2023). The risk of retinal vein occlusion among patients with neovascular age related macular degeneration: a large-scale cohort study. Eye (Lond). 37 (7), 1445–1450. 10.1038/s41433-022-02163-7 35778605 PMC10170074

[B45] Wishart D. S. Feunang Y. D. Guo A. C. Lo E. J. Marcu A. Grant J. R. (2018). DrugBank 5.0: a major update to the DrugBank database for 2018. Nucleic Acids Res. 46 (D1), D1074–D1082. 10.1093/nar/gkx1037 29126136 PMC5753335

[B46] Wu J. Zheng Z. Chong Y. Li X. Pu L. Tang Q. (2018). Immune responsive release of Tacrolimus to overcome organ transplant rejection. Adv. Mater 30 (45), e1805018. 10.1002/adma.201805018 30255648

[B47] Wu S. N. Chen X. D. Yan D. Wang Y. Q. Wang S. P. Guan W. Y. (2024a). Drug-associated glaucoma: a real-world study based on the Food and Drug Administration adverse event reporting system database. Clin. Exp. Ophthalmol. Published online October 25. 10.1111/ceo.14454 39460378

[B48] Wu S. N. Chen X. D. Zhang Q. H. Wang Y. Q. Yan D. Xu C. S. (2024b). Drug-related keratitis: a real-world FDA adverse event reporting system database study. Transl. Vis. Sci. Technol. 13 (9), 17. 10.1167/tvst.13.9.17 PMC11421680 39287587

[B49] Wu S. N. Huang C. Wang Y. Q. Chen X. D. Li X. Zhang S. Q. (2024c). Real-World large sample assessment of drug-related dry eye risk: based on the FDA adverse event reporting system database. Asia Pac J. Ophthalmol. (Phila) 13 (5), 100104. 10.1016/j.apjo.2024.100104 39343068

[B50] Yin Y. Shu Y. Zhu J. Li F. Li J. (2022). A real-world pharmacovigilance study of FDA Adverse Event Reporting System (FAERS) events for osimertinib. Sci. Rep. 12 (1), 19555. 10.1038/s41598-022-23834-1 36380085 PMC9664039

[B51] Yu R. J. Krantz M. S. Phillips E. J. Stone C. A. (2021). Emerging causes of drug-induced anaphylaxis: a review of anaphylaxis-associated reports in the FDA adverse event reporting system (FAERS). J. Allergy Clin. Immunol. Pract. 9 (2), 819–829.e2. 10.1016/j.jaip.2020.09.021 32992044 PMC7870524

[B52] Yusuf I. H. Lotery A. J. Ardern-Jones M. R. (2018). Joint recommendations for retinal screening in long-term users of hydroxychloroquine and chloroquine in the United Kingdom, 2018. Br. J. Dermatol 179 (4), 995–996. 10.1111/bjd.16782 29770429

[B53] Zhao H. Li Z. R. Zhang Q. Zhong M. K. Yan M. M. Qiu X. Y. (2023). Sodium-glucose co-transporter-2 inhibitor (SGLT2i) treatment and risk of osteomyelitis: a pharmacovigilance study of the FAERS database. Front. Pharmacol. 14, 1110575. 10.3389/fphar.2023.1110575 36865915 PMC9971937

[B54] Zhao J. Tao Y. (2024). Adverse event reporting of the IGF-1R monoclonal antibody teprotumumab: a real-world study based on the US food and drug administration adverse event reporting system. Front. Pharmacol. 15, 1393940. 10.3389/fphar.2024.1393940 39185318 PMC11341477

