# Supplementary material for: Drug-induced retinal vein occlusion: a disproportionality analysis from the FDA adverse event reporting system (2004–2023)
Source: Front Pharmacol. 2024 Dec 13;15:1480269. doi: 10.3389/fphar.2024.1480269 (PMC11671269; doi:10.3389/fphar.2024.1480269)
Supplement: Supplementary file 1 [file Table1.docx]

**Supplementary Table 1. Drugs associated with RVO categorized by BCPNN risk levels.**

| Drug | n | Classification | BCPNN value | Risk degree |
| --- | --- | --- | --- | --- |
| Mirabegron | 3 | Adrenergic Receptor Agonists | 4.64 | +++ |
| Raloxifene | 26 | Hormone Modulators | 4.37 | +++ |
| Tadalafil | 14 | PDE5 Inhibitors | 3.488 | +++ |
| Fingolimod | 6 | Immunomodulators | 3.215 | +++ |
| Bimatoprost | 4 | Prostaglandin Analogs | 3.065 | ++ |
| Brimonidine | 3 | Adrenergic Receptor Agonists | 2.985 | ++ |
| Sildenafil | 47 | PDE5 Inhibitors | 2.956 | ++ |
| Vardenafil | 3 | PDE5 Inhibitors | 2.775 | ++ |
| Verteporfin | 4 | Photosensitizers | 2.626 | ++ |
| Celecoxib | 4 | Non-Steroidal Anti-Inflammatory Drugs | 2.618 | ++ |
| Sorafenib | 4 | Kinase Inhibitors | 2.609 | ++ |
| Anastrozole | 7 | Hormone Modulators | 2.285 | ++ |
| Drospirenone | 11 | Hormone Modulators | 1.763 | ++ |
| Upadacitinib | 8 | Immunomodulators | 1.719 | ++ |
| Hydroxychloroquine | 3 | Immunomodulators | 1.65 | ++ |
| Encorafenib | 3 | Kinase Inhibitors | 1.57 | ++ |
| Ponatinib | 7 | Kinase Inhibitors | 1.487 | + |
| Peginterferon beta-1a | 3 | Immunomodulators | 1.392 | + |
| Aripiprazole | 5 | Neurotransmitter Modulators | 1.253 | + |
| Estradiol | 6 | Hormone Modulators | 1.193 | + |
| Letrozole | 4 | Hormone Modulators | 1.027 | + |
| Vemurafenib | 10 | Kinase Inhibitors | 0.937 | + |
| Dabrafenib | 4 | Kinase Inhibitors | 0.602 | + |
| Tacrolimus | 7 | Immunomodulators | 0.463 | + |
| Rofecoxib | 47 | Non-Steroidal Anti-Inflammatory Drugs | 0.19 | + |

**Note:** Risk levels based on BCPNN values: + (Low, 0-1.5), ++ (Moderate, 1.5-3), +++ (High, >3).
